# Research on the Application of Artificial Neural Network-Based Virtual Image Technology in College Tennis Teaching

**DOI:** 10.1155/2022/4935121

**Published:** 2022-07-08

**Authors:** Ruizhe Hu, Xiaocan Cui

**Affiliations:** School of Physical Education (Main Campus), Zhengzhou University, Zhengzhou 450001, Henan, China

## Abstract

At the same time that my country has shifted from high-speed development to high-quality development, my country has also put forward new requirements for education development. Due to the limited study time during college, each student's study habits and learning process are also different, and the degree of connection between tennis lessons is high, so there will be polarization when learning tennis. With the development of science and technology, more and more technological innovations are integrated into the classroom, and traditional teaching methods can no longer keep up with the pace of the times. Tennis teaching is a subject of equal proportion between theory and practice. The traditional teaching method simplifies the theory, which makes students to have some bad phenomena when they practice. Aiming at this series of problems, this paper uses algorithms such as softmax function and threshold function to construct an application model of virtual image technology based on the artificial neural network in tennis teaching. The research results of the article show that: (1) the average accuracy rate of the method in this paper is 97.22%, and the highest accuracy rate is 99.17%. The average accuracy rate also tends to increase with the increase of sample size; the recall rate is the highest, and the highest recall rate is 99.36%. The average recall rate is 96.77%; the highest correct rate is close to 100% and is significantly higher than the other three methods; the average correct rate reaches 98.8%; the response time is the shortest; the average response time is 33 ms; and the response time increases with the increase of the sample size. (2) After using this model, tennis skills have been improved, with an average of 12 in situ flips, an average of 7 in situ rackets, an average of 5 in situ forehand draws, and an average of 3 in situ backhand draws. (3) The average forehand and backhand scores of the class after the experiment were 90 and 86; the average forehand and backhand stability were 8 and 7; and the average forehand and backhand accuracy were 31 and 29, respectively. The average depth of forehand and backhand is 36 and 32. (4) Most of the students are satisfied with this model, and they all choose to strongly agree and relatively agree, and the percentage of very agree that helps stimulate learning has reached 60.52%, and no students choose to disagree very much.

## 1. Introduction

At present, although the traditional teaching methods of tennis in colleges and universities are helpful for students to understand and master the basic tennis movements, the traditional methods are relatively simple and cannot provide deeper guidance to students. Therefore, this paper uses the softmax function and threshold function to construct the application model of virtual image technology based on an artificial neural network in tennis teaching. Improve students' learning ability of tennis through virtual imaging technology. This paper has received a lot of support on the basis of previous results. Artificial neural networks solve many problems that are difficult for modern computers to solve in the field of model recognition [1]. It exhibits good intelligence properties [2]. Artificial neural networks are proposed in the technology of modern neuroscience research results [3]. Neuron processing units can represent different objects [4]. Artificial neural networks enable parallel distributed systems [5]. It adopts a completely different mechanism from traditional artificial intelligence and information processing technology [6]. And it has the characteristics of self-adaptation, self-organization, and real-time learning [7]. Virtual is a description of distance and impossibility [8]. The most favorable narrative method of digital images is its virtuality [9]. Virtual imaging technology uses information as material and transforms information into perceived reality [10]. Virtual can merge the vast and the subtle into one [11]. Virtual image technology combines art design and computer technology [12]. Virtual imaging technology is a general term for existing virtual reality, augmented reality, and projection technologies [13]. The artificial neural network model mainly considers the topology of network connections [14]. An artificial neural network is a nonlinear, adaptive information processing system composed of a large number of interconnected processing units [15].

## 2. Basic Knowledge

### 2.1. Artificial Neural Network

#### 2.1.1. Neural Network Model

**Figure 1:**
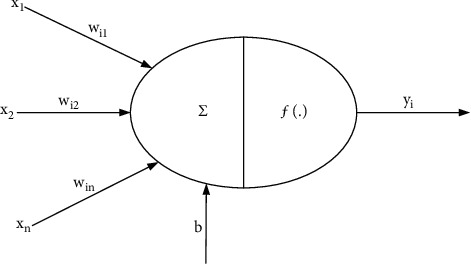
Artificial neural network model.

Artificial Neural Network (ANN for short) [16] is an information processing system established by humans based on the understanding of the operating rules of the brain neural network. The system can imitate the structure of the brain neural network to achieve certain functions. A neural network is an adaptive nonlinear dynamic system composed of multiple linear and nonlinear neurons connected to each other according to a certain method, in which neurons are the storage and processing units of information, and the learning process of the neural network is based on the input between neurons. The pattern connection weights are continuously adjusted to form a memory for the input pattern. A simple neuron model is shown in Figure 1.

In Figure 1, the input of the neuron is represented by *X*=(*x*_1_, *x*_2_, *x*_3_,…, *x*_*n*_); the corresponding weight value of each input is represented by *W*=(*w*_1_, *w*_2_, *w*_3_,…, *w*_*n*_); the bias value of the neuron is represented by *b*; the activation function of the neuron is represented by *f*(*·*); and the input of the neuron is represented by *y*.

(1) $$\begin{array}{c} \cdot =\sum_{i=1}^{n}w_{i}x_{i}+b, \end{array}$$

(2) $$\begin{array}{c} y=f\left(\cdot \right). \end{array}$$

#### 2.1.2. Neuron Activation Function

The activation function is the main processing method of artificial neural network information. The activation functions proposed according to different research problems can be divided into three categories:

(1) Linear activation function: the main function of the linear activation function is to linearly process the input information of the neuron, that is, the output information and the input information satisfy a linear relationship. The most common linear activation function is the pure-line activation function [17]. The function expression is

(3) $$\begin{array}{c} f\left(x\right)=kx. \end{array}$$

(2) Nonlinear activation function: the main function of the nonlinear activation function is to nonlinearly process the input information of the neuron. The two activation functions of Sigmoid and Tanh are mainly used. The function expressions are as follows:

Sigmoid function:

(4) $$\begin{array}{c} f\left(x\right)=\frac{1}{1+e^{-x}}. \end{array}$$

Tanh function:

(5) $$\begin{array}{c} f\left(x\right)=\frac{1-e^{-x}}{1+e^{-x}}. \end{array}$$

(3) Threshold function: the threshold function adopts a step function, and the output of the neuron is a binary variable [18]. Its function expression is

(6) $$\begin{array}{c} f\left(x\right)=\left\{\begin{array}{cc} 1, & x\geq 0\\ 0, & x<0 \end{array}\right\}. \end{array}$$

(4) Softmax function: the softmax function is defined as follows:

(7) $$\begin{array}{c} S_{i}=\frac{e^{y_{i}}}{\sum_{j}e^{y_{i}}}. \end{array}$$

The softmax function shows the concept of mutual exclusion, that is, the closer the judgment result is to a certain class, the closer the corresponding value of this class is to 1, and the closer to 0 for other classes.

(5) Gaussian error linear unit: the Gaussian error linear unit (GELU) adjusts the output value through a gating mechanism, defined as follows:

(8) $$\begin{array}{c} GELU\left(x\right)=xP\left(X\leq x\right), \end{array}$$

where *P*(*X* ≤ *x*) is the cumulative distribution function of the Gaussian distribution.

The Gaussian error linear unit is approximated by the Tanh function:

(9) $$\begin{array}{c} GELU\left(x\right)\approx 0.5x\left(1+\tanh\left(\sqrt{\frac{2}{\pi }}\left(x+0.044715x^{3}\right)\right)\right). \end{array}$$

Approximate using the logistic function:

(10) $$\begin{array}{c} GELU\left(x\right)\approx xL\left(1.702x\right). \end{array}$$

#### 2.1.3. Loss Function

The loss function is usually used to describe the difference between the prediction result of the model on the sample value and the actual value, which represents the accuracy of the model to a certain extent. The loss function includes the mean square error function and the cross-entropy function.

(1) Mean square error function: the mean square error function uses the mean square error in statistics to characterize the deviation of the predicted value from the actual value and is defined as follows:

(11) $$\begin{array}{c} \text{Loss}=\frac{\sum {\left(y-y^{\prime }\right)}^{2}}{n}, \end{array}$$

where *y* is the label value, *y*′ is the predicted value, and *n* is the number of samples.

(2) Cross-entropy function: the cross-entropy function represents the deviation between two probability steps. Assuming that both *p* and *q* represent the law of probability, the cross entropy of *p* represented by *q* is

(12) $$\begin{array}{c} H\left(p,q\right)=-\sum_{x}p\left(x\right)\log_{2}q\left(x\right). \end{array}$$

### 2.2. Artificial Neural Network Algorithm

The learning process of the artificial neural network is composed of forward propagation and back propagation [19]. The algorithm process is as follows.

Initialize weights and thresholds [20]: after the network BP (*n*, *q*, *m*) is determined, the network algorithm includes the weight from the *i* unit of the input layer to the *j* unit of the hidden layer, the weight of *W*_*ij*_^1^(*i*=1,…, *n*; *j*=1,…, *k*) from the *j* unit of the hidden layer to the *k* unit of the output layer, and the weight of *W*_*ij*_^0^(*j*=1,…, *q*; *k*=1,…, *m*). The activation threshold of the *j* unit of the hidden layer is *θ*_*j*_^*H*^(*j*=1,…, *q*), and the activation threshold of the *j* unit of the output layer is *θ*_*k*_^0^(*k*=1,…, *m*). The above weights and initial thresholds are randomly generated before the network is trained.

Training sample information [21]: assuming that *P* is a common training sample, input the *r*(*r*=1,…, *p*) training sample information to the first hidden layer of forward propagation and obtain the output information of the hidden layer through the action of the activation function *f*(*x*).

(13) $$\begin{array}{c} H_{jr}=f\left(\sum_{i=1}^{n}w_{ij}^{1}x_{ir}\theta_{j}^{1}\right),\;\left(j=1,\ldots ,q;r=1,\ldots ,p\right). \end{array}$$

The hidden layer output information is transmitted to the output layer, which may be the final output result [22].

(14) $$\begin{array}{c} Y_{kr}=f\left(\sum_{j=1}^{q}w_{jk}^{0}H_{jr}\theta_{k}^{0}\right),\;\left(k=1,\ldots ,m;r=1,\ldots ,p\right). \end{array}$$

In the process of forward propagation of learning information in the network, the error of another process is backpropagated [23]. If there is an error between the network output and the desired output value, then backpropagate the error, using all adjusted network weights and thresholds.

(15) $$\begin{array}{c} \Delta w\left(t+1\right)=\eta \frac{\partial E}{\partial w}\alpha \Delta w\left(t\right), \end{array}$$

where Δ*w*(*t*) is the modified value of the *t* training weight and threshold [24] and *η* and *α* are the scale factor and momentum factor, respectively.

(16) $$\begin{array}{c} E=\frac{1}{2}\sum_{k=1}^{m}\sum_{r=1}^{p}{\left(Y_{r}-t_{r}\right)}^{2}. \end{array}$$

The above two procedures are repeated until the error between the network outputs reaches a certain requirement and desired output.

### 2.3. The Basic Principle of Artificial Neural Network

The input net_*j*_^*m*^ for the *j* neuron in layer *m* is

(17) $$\begin{array}{c} {\text{net}}_{j}^{m}=\sum_{i=1}^{n}w_{ij}^{m-1}o_{i}^{m-1}+b_{j}^{m}. \end{array}$$

Then, the output *o*_*j*_^*m*^ of the *j* neuron in the *m* layer is

(18) $$\begin{array}{c} o_{j}^{m}=f_{j}^{m}\left({\text{net}}_{j}^{m}\right) \end{array}$$

(19) $$\begin{array}{c} o^{n}=f^{n}\left(w^{n-1}f^{n-1}\frac{\left(\ldots \left(w^{i+1}f^{i+1}\left(w^{i}o^{i}+b^{i+1}\right)\ldots \right)\right)}{n-2}+b^{n-1}\right)+b^{n}, \end{array}$$

where *w*_*ij*_^*m*−1^ is the weight between the *i* neuron in the *m* − 1 layer and the *j* neuron in the *m* layer, *b*_*j*_^*m*^ is the bias value of the *j* neuron in the *m* layer, and *o*_*i*_^*m*−1^ is the second layer. Three neurons were applied to output; *f*_*j*_^*m*^() is the activation function of the *j* neuron in the *m* layer.

Thus, the expression of the final output information of the neural network about the input information can be obtained. The expression is represented by a vector as follows:

(20) $$\begin{array}{c} o^{n}=f^{n}\left(w^{n-1}f^{n-1}\left(w^{n-2}o^{n-2}+b^{n-1}\right)+b^{n}\right). \end{array}$$

### 2.4. Learning Rules of Artificial Neural Networks

The error function generated for sample *a* and the *h* output neuron is defined as follows:

(21) $$\begin{array}{c} e_{h}=d_{h}-o_{h}. \end{array}$$

In order to make the partial derivative continuous, a quadratic error function is generally used, and the total error function for sample *a* is

(22) $$\begin{array}{c} E=\frac{1}{2}\sum_{h}^{L}{\left(d_{h}-o_{h}\right)}^{2}. \end{array}$$

When training, when *p* training sample is input at a time, the total error function is

(23) $$\begin{array}{c} E_{p}=\frac{1}{2}\sum_{p=1}^{p}\sum_{h=1}^{L}{\left(d_{h}^{p}-o_{h}^{p}\right)}^{2}. \end{array}$$

When using the fastest gradient descent algorithm [25]. The formula is

(24) $$\begin{array}{c} w_{ij}^{m}\left(k+1\right)=w_{ij}^{m}\left(k\right)-lr\frac{\partial {\hat{E}}_{p}}{\partial w_{ij}^{m}}, \end{array}$$

(25) $$\begin{array}{c} b_{j}^{m}\left(k+1\right)=b_{j}^{m}\left(k\right)-lr\frac{\partial {\hat{E}}_{p}}{\partial b_{j}^{m}}, \end{array}$$

where *w*_*ij*_^*m*^(*k*+1) and *w*_*ij*_^*m*^(*k*) are the weight values between the *i* neuron in the *m* − 1 layer and the *j* neuron in the *m* layer in the *k*+1 and *k* iterations, respectively; *b*_*j*_^*m*^(*k*+1) and *b*_*j*_^*m*^(*k*) are the *k*+1 and the bias value of the *j* neuron in the *m* layer at the *k* iteration, respectively; *lr* is the learning rate; and ${\hat{E}}_{p}$ is the approximate error at the *k* iteration.

## 3. Application of Virtual Image Technology in College Tennis Teaching

### 3.1. Overview of Virtual Imaging Technology

#### 3.1.1. Concept of Virtual Image Technology

The virtual image is a visual technology and art based on digital technology. Virtual imaging technology is a combination of photography technology, projection technology, display technology, and other technologies, which can help people better understand the known real space and the unknown virtual space. It simulates and reorganizes text, images, and other information through digital technology to form abstract or real spatial scenes with interactivity, virtuality, and immersion.

#### 3.1.2. Classification of Virtual Imaging Technology

Virtual imaging technology can be divided into virtual reality technology, augmented reality technology, holographic projection technology, fog screen stereo imaging technology, wall projection technology, and interactive projection technology as shown in Table 1.

#### 3.1.3. Technical Characteristics of Virtual Images

Virtual imaging technology has the characteristics of integration, fidelity, immersion, imagination, artistry, and interaction as shown in Table 2.

### 3.2. Basic Principles of Tennis Teaching

Tennis teaching has the principle of health, which means that the physical safety of students should be guaranteed first in the tennis teaching process; in the process of tennis teaching, the teaching content should be gradual and the principle of low level to high level, starting from the actual principle, from simple to complex and gradually improve. According to the students' physical load and practice venues to arrange teaching, students consciously and actively participate in learning activities; students should firmly grasp the knowledge of all aspects of tennis.

### 3.3. Model Construction

**Figure 2:**
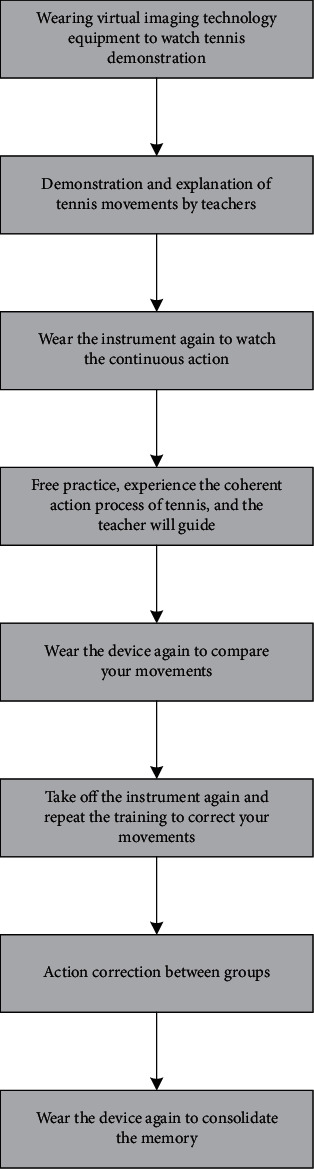
Tennis teaching model.

In this paper, a model of college tennis teaching based on virtual image technology based on an artificial neural network is constructed, as shown in Figure 2. The tennis teaching model in colleges and universities is divided into three steps: the first step is audition learning, the second step is practical mastery, and the third step is interactive analysis.

In the first step in tennis teaching, students first need to wear virtual imaging technology equipment to gain a preliminary understanding of tennis teaching through the equipment and then take off the equipment, and the teacher will demonstrate and explain the standard movements; after the teacher's explanation, the students need to wear the equipment again and watch a continuous tennis movement to speed up their understanding of the action.

In the second step, the students take off the instrument again and practice freely. The teacher guides the students' movements during this process; after the teacher finds the students' problems, the students wear the instruments again to compare their movements to find their own problems; Remove the instrument again, repeat the previous practice, and correct the problems found in the process; and the teacher also corrects the problems of the students in the process.

Finally, in the third step, students form groups to practice and supervise each other and wear the instrument again to consolidate the practice.

## 4. Experimental Analysis

### 4.1. Model Testing

In this paper, an application model of artificial neural network-based virtual image technology in college tennis teaching is constructed, and algorithms such as softmax function and threshold function are used in the model. First, the model will be tested, and the advantages of the model will be checked by comparing it with BP neural network, deep neural network, and traditional methods, and whether it meets the requirements.

In the model testing stage, in order to test the accuracy, recall, correctness, and response time of the model under different sample sizes, the standard degree of students' actions in the learning process was selected as the research object. The experimental sample sizes were divided into 6 groups, namely 10, 30, 50, 70, 90, and 110.

The criteria for the model to meet the requirements are as follows: the average accuracy rate of the test samples is more than 97%, the average recall rate is greater than 96%, the average accuracy rate is greater than 98%, and the average response time is less than 34%; then the model meets the requirements.

**Figure 3:**
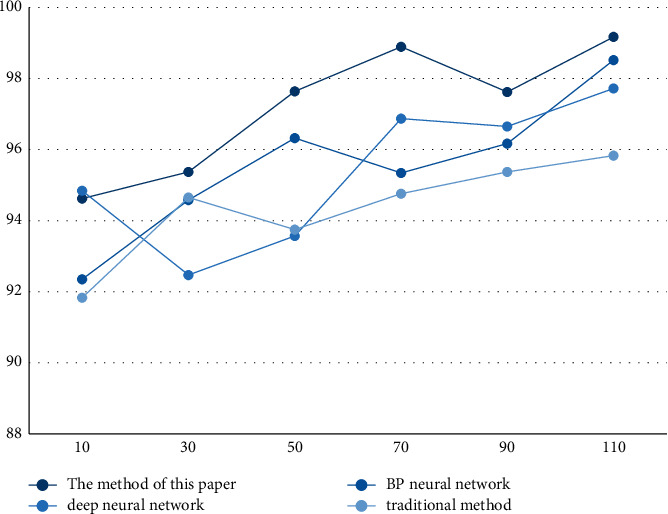
Accuracy.

According to the results in Figure 3 and Table 3, the average accuracy rate of the method in this paper is 97.22%, and the highest accuracy rate is 99.17%. The average accuracy rate also tends to increase with the increase of the sample size, indicating that the method in this paper meets the requirements. And, by comparing the method in this paper with the other three methods, it can be seen that the method in this paper has obvious advantages and the highest accuracy.

**Figure 4:**
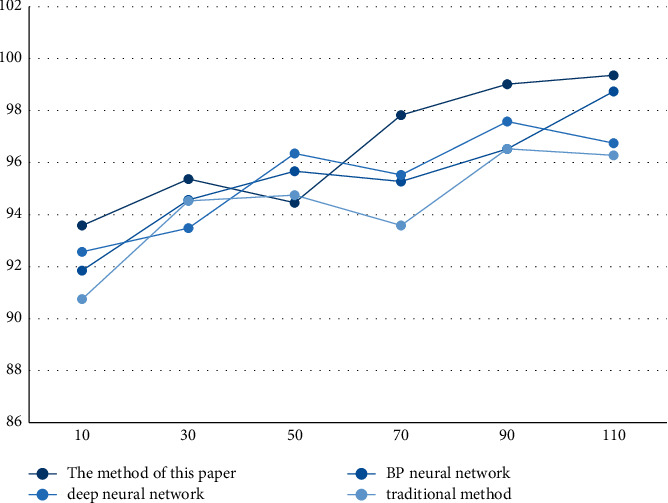
Recall rate.

As can be seen from Table 4, the recall rate of the method in this paper is the highest; the highest recall rate is 99.36%; and the average recall rate is 96.77%, which meets the inspection standard. According to Figure 4, it can be clearly seen that the overall recall rate is on the rise, and it is concentrated between 94% and 96%.

**Figure 5:**
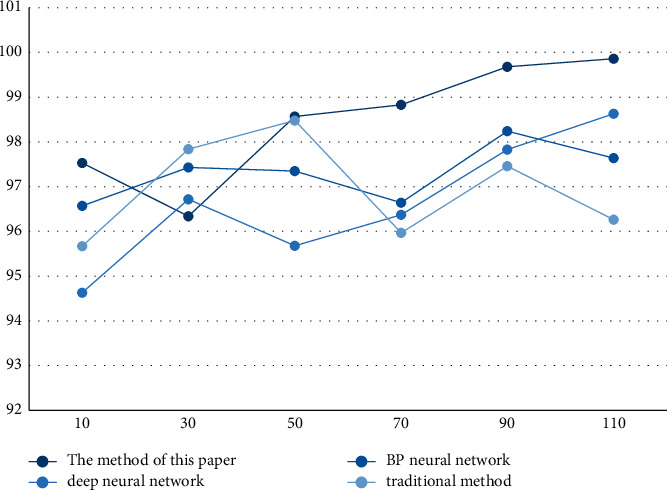
Correct rate.

The results in Figure 5 and Table 5 show that the highest accuracy rate of the method in this paper is close to 100%, which is significantly higher than that of the other three methods, and the average accuracy rate reaches 98.8%, indicating that the method in this paper meets the requirements. On the whole, the correct rate of the four methods is above 94%; the average correct rate of the BP neural network is 97.3%; the average correct rate of the deep neural network is 96.6%; and the average correct rate of the traditional method is 96.9%.

**Figure 6:**
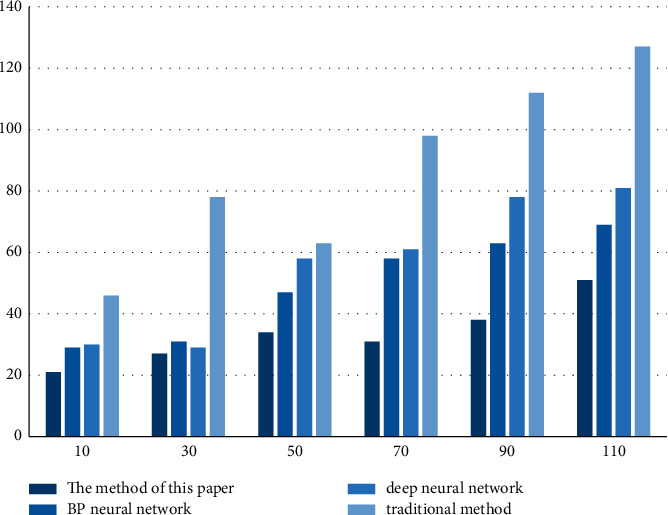
Response time.

The experimental results in Figure 6 and Table 6 show that the method in this paper requires the shortest response time, with an average response time of 33 ms, and the response time increases with the increase of the sample size. The traditional method had the highest response time, which was twice as high as the other methods with a sample size of 30.

It can be seen from the experimental results that the method in this paper has reached the standard in terms of average precision, average recall, average correct rate, and average response time and meets the requirements. Compared with the other three methods, the method has obvious advantages and is feasible.

### 4.2. Experimental Simulation

After the model meets the test criteria, it will be used in formal teaching. In order to test the students' learning effect under the model, the students' learning results will be compared with those before the experiment.

#### 4.2.1. Comparison of Student Tennis Skills

**Figure 7:**
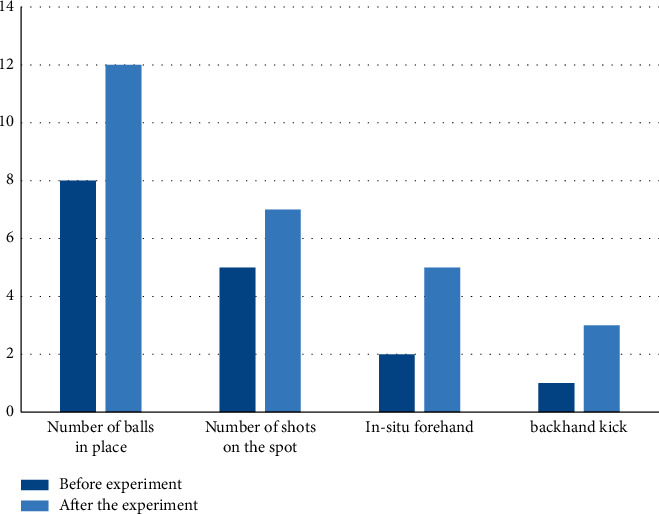
Comparison of student tennis skills.

Students in one class were randomly selected to conduct the experiment, and their average number of in situ hits, average in situ rackets, average in situ forehand draws, and average in situ backhand draws were recorded before and after the experiment. The experimental results are shown in Figure 7.

From the results in Figure 7, it can be seen that the tennis skills of the students before the experiment were relatively weak. After using the model, the tennis skills were improved. The average number of in situ flips was 12 times; the average number of in situ racquets was 7; and the average in situ strokes were 7 times. There is an average of five forehand draws and three backhand draws.

#### 4.2.2. Comparison of Forehand and Backhand Performance

Forehand and backhand assessment, stability, accuracy, and depth of stroke are also important components of tennis assessment in tennis teaching.

**Figure 8:**
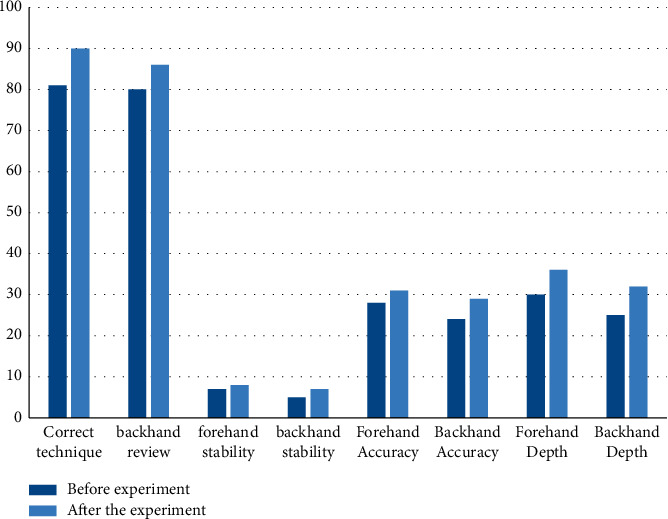
Comparison of forehand and backhand scores.

Forehand and backhand skill evaluation is to score students' actions during the hitting process; forehand and backhand stability is based on students hitting the ball 10 times with forehand and backhand, respectively, and the number of hits is the final score. Forehand and backhand accuracy: it means that each student hits the ball 10 times alternately within the specified different score areas and hits the ball to get the corresponding score; the depth of forehand and backhand hitting is the depth area corresponding to each student's different scores.  Figure 8 shows the average scores of the students in this class before and after using the model in this paper:

The results in Figure 8 show that the average forehand and backhand scores of the class before the experiment were 81 and 80, the average forehand and backhand stability were 7 and 5, and the average forehand and backhand accuracy were 28 and 24, respectively. Forehand and backhand depths are 30 and 25. The average forehand and backhand scores of the class after the experiment were 90 and 86 points, respectively; the average forehand and backhand stability were 8 and 7 times, respectively; and the average forehand and backhand accuracy were 31 points and 29 points, with an average forehand and backhand depth of 36 and 32 points, respectively. And the scores of the students in this class have improved after using the model, indicating that the model has obvious advantages in improving students' performance.

### 4.3. Model Evaluation

**Figure 9:**
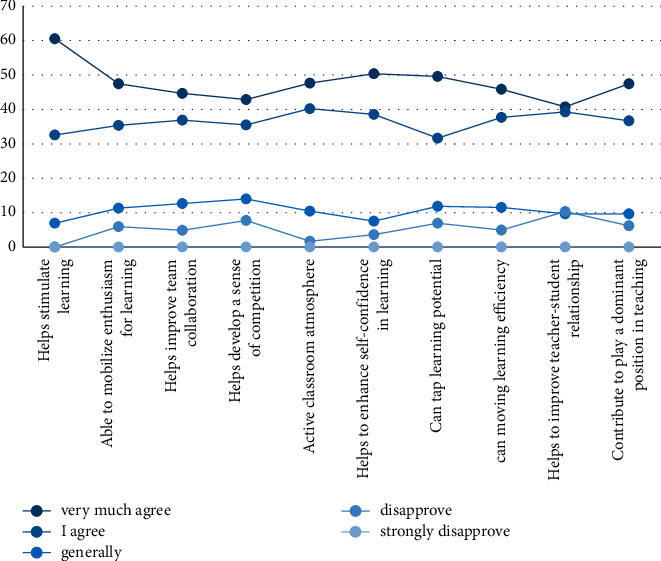
Model evaluation.

After the experiment, the classmates' evaluation of the tennis teaching model using virtual image technology was statistically analyzed by means of questionnaires. It is evaluated in 7 aspects: to cultivate a sense of competition, activate the classroom atmosphere, enhance self-confidence in learning, tap learning potential, improve learning efficiency, enhance teacher-student emotion, and play a dominant role in teaching. The evaluation results are shown in Figure 9.

It can be seen from Figure 9 that most of the students are satisfied with this model, and they all choose to strongly agree or agree, indicating that the students have a high degree of recognition of the tennis teaching model using virtual image technology. And the percentage of very agreeable to help stimulate learning reached 60.52%, and no classmates chose to be very disapproved.

## 5. Conclusion

College physical education is an important part of the teaching process in colleges and universities, and tennis teaching is a subject that combines theory and practice, so the teaching mode of tennis is different from that of other subjects. With the development of the times, the traditional tennis teaching method has a certain lag in the teaching process. Therefore, this paper uses various algorithms such as softmax function and threshold function to construct a virtual image technology based on an artificial neural network in tennis teaching. Improve students' tennis skills through a combination of tennis teaching and virtual technology.

The findings of the article show that

1. The average accuracy rate of the method in this paper is 97.22%, and the highest accuracy rate is 99.17%. The average accuracy rate also tends to increase with the increase of the sample size, indicating that the method in this paper meets the requirements.
2. The recall rate of the method in this paper is the highest; the highest recall rate is 99.36%; and the average recall rate is 96.77%, which meets the inspection standard.
3. The highest accuracy rate of the method in this paper is close to 100%, which is significantly higher than that of the other three methods, and the average accuracy rate reaches 98.8%, indicating that the method in this paper meets the requirements.
4. The response time required by the method in this paper is the shortest, with an average response time of 33 ms, and the response time increases with the increase of the sample size.
5. The tennis skills of the students before the experiment were relatively weak. After using the model, the tennis skills were improved. The average number of in situ flips was 12; the average number of in situ rackets was 7; and the average in situ forehand was drawn. The ball is 5 times, and the average backhand kick is 3 times.
6. The scores of the students in this class have improved after using the model, indicating that the model has obvious advantages in improving students' performance.
7. Most of the students are satisfied with this model, and they all choose to strongly agree or agree, indicating that the students have a high degree of recognition of the tennis teaching model using virtual imaging technology.

According to the experimental results, in the following teaching process, students' interest in learning and autonomous learning ability should be strengthened; teachers' own teaching ability professional level should be improved; an open and free learning division should be created to increase students' interest in the class. Although the artificial neural network-based virtual image technology constructed in this paper has obvious advantages in the application model experimental results of tennis teaching, there are still many shortcomings and certain limitations. The model constructed in this paper is limited to tennis teaching. It is hoped that in the following research, researchers can conduct in-depth research on the scope of application so that the model is not limited to tennis teaching and increases the scope of application of the model.

## Figures and Tables

**Table 1:** Classification of virtual imaging technology.

Classification	Content
Virtual reality (VR)	Virtual reality technology is a general term for a technical system used to establish a virtual environment for the experiencer to observe and interact with. Use 3dmax, Unity3d, and other software to model and create virtual scenes, capture and simulate human movement through hardware facilities such as Kinect and Arduino, and interact with virtual scenes; generate instructions through C language and Java programming; and form feedback.

Augmented reality (AR)	Augmented reality technology will recognize and analyze images observed with the camera; classify and identify matching images, images, and other digital information in a database; and display it on the screen overlaid with the actual scene.

Holographic projection technology	Holographic imaging technology projects the image onto the actual environment or transparent medium without the audience wearing the device, creating the illusion that the image is suspended in the air. Then, interact with the image through the interactive device.

Fog curtain stereo imaging technology	Fog screen stereo imaging technology uses a spray device instead of a traditional projection screen to generate an artificial water mist wall and generates a flickering fog screen projection image through an electric device to form a holographic image.

Wall projection technology	Wall projection technology is mainly used for the outer panels of urban buildings. The urban exterior wall projection is mainly composed of a laser projector, a screen segmentation control matrix technology, and an exterior wall laser projection system composed of an intelligent central control system.

Interactive projection technology	Interactive projection technology is a projection system composed of projection equipment, infrared sensors, motion capture equipment, and computers. The captured data is analyzed using infrared cameras and sensors to track and identify data such as body movements and the voice of the experiencer. Combined with real-time image interaction tracking, it produces real-time interaction effects between participants and projects.

**Table 2:** Technical characteristics of virtual images.

Feature	Content
Integration	Virtual image technology is the product of technology development and traditional images. Virtual image technology is a new image technology supported by projection technology, display technology, sensing technology, and other technologies.

Fidelity	Virtual imaging technology is to reproduce reality through the current simulation or focus on the current image; contrary to the real optical illusion, the user's visual authenticity and the authenticity of the image are unified. There are many real experiences in real vision, intelligence, vision, and relationships.

Immersion	In fact, outside of the ideal visual image, people are really confused about pain. The combined behavior of public experience and sensory psychological factors creates an illusion of interest in the user's real environment. Through sensory technology and real-time, users can connect to virtual environments to create physical and mental experiences.

Imaginative	Virtual images not only can reproduce the real environment but also can imagine an illustrative environment, satisfying the curiosity as well as the visual and psychological needs of the experiencer. The creative process of virtual image technology is special, so it can be integrated into the imagination of the creative stage, increase the scale of the work, and increase the scope of human knowledge and imagination.

Artistry	The production of virtual image content is people's thinking and behavior, and art is also designed and used based on people's main aesthetic preferences, emotions, thinking, and behavior.

Interactivity	Through the recognition of the experienced expressions and sounds, the information of body movement is captured and temporarily returned in real time, and the interaction of images is formed.

**Table 3:** Accuracy (%).

Model	10	30	50	70	90	110
The method of this paper (%)	94.62	95.37	97.64	98.89	97.62	99.17
BP neural network (%)	92.35	94.58	96.32	95.34	96.17	98.52
Deep neural network (%)	94.84	92.47	93.57	96.87	96.65	97.72
Traditional method (%)	91.83	94.65	93.75	94.76	95.37	95.83

**Table 4:** Recall rate (%).

Model	10	30	50	70	90	110
The method of this paper (%)	94.58	95.37	94.46	97.83	99.02	99.36
BP neural network (%)	91.85	94.57	95.67	95.27	96.53	98.74
Deep neural network (%)	92.57	93.48	96.35	95.53	97.58	96.75
Traditional method (%)	90.75	94.53	94.75	93.58	96.53	96.28

**Table 5:** Correct rate (%).

Model	10	30	50	70	90	110
The method of this paper (%)	97.53	98.34	98.57	98.83	99.68	99.86
BP neural network (%)	96.57	97.43	97.35	96.64	98.24	97.64
Deep neural network (%)	94.63	96.72	95.68	96.37	97.83	98.63
Traditional method (%)	95.67	97.84	98.48	95.97	97.46	96.26

**Table 6:** Response time (ms).

Model	10	30	50	70	90	110
The method of this paper (ms)	21	27	34	31	38	51
BP neural network (ms)	29	31	47	58	63	69
Deep neural network (ms)	30	29	58	61	78	81
Traditional method (ms)	46	78	63	98	112	127

## Data Availability

The experimental data used to support the findings of this study are available from the corresponding author upon request.

## References

[1] Katz W. T., Snell J. W., Merickel M. B. (1992). [29] Artificial neural networks. *Methods in Enzymology*.

[2] Govindaraju R. S. (2000). Artificial neural networks in hydrology. *Journal of Hydrologic Engineering*.

[3] Dalziel B. D., Morales J. M., Fryxell J. M. (2008). Fitting probability distributions to animal movement trajectories: using artificial neural networks to link distance, resources, and memory. *The American Naturalist*.

[4] Andrews R., Diederich J., Tickle A. B. (1995). Survey and critique of techniques for extracting rules from trained artificial neural networks. *Knowledge-Based Systems*.

[5] Silverman D., Dracup J. A. (2000). Artificial neural networks and long-range precipitation prediction in California. *Journal of Applied Meteorology*.

[6] White H. (1989). Learning in artificial neural networks: a statistical perspective. *Neural Computation*.

[7] Xin Y. (2010). A review of evolutionary artificial neural networks. *International Journal of Intelligent Systems*.

[8] Linte C. A., White J., Eagleson R., Guiraudon G. M., Peters T. M. (2010). Virtual and augmented medical imaging environments: enabling technology for minimally invasive cardiac interventional guidance. *IEEE Reviews in Biomedical Engineering*.

[9] Buckland J. (2013). Imaging technology acts as a ‘virtual skin biopsy’ in SSc. *Nature Reviews Rheumatology*.

[10] Kitaoka H., Tamura S. (2000). Virtual imaging of the lung based on computational morphology. *Medical imaging technology*.

[11] Wu Y., Chen Y. (2021). Influence of virtual imaging technology based on Html5 technology on digital painting. *Microprocessors and Microsystems*.

[12] Leong F., Graham A. K., Mcgee J. O. (2000). Virtual histological imaging utilising next generation telepathology technology. *The Journal of Pathology*.

[13] Han X., Yang Q. (2014). Construction and application of the network-based platform for virtual practice teaching of medical imaging technology specialty in higher vocational education. *China Medical Education Technology*.

[14] Szkuta B. R., Sanabria L. A., Dillon T. S. (1999). Electricity price short-term forecasting using artificial neural networks. *IEEE Transactions on Power Systems*.

[15] Sietsma J., Dow R. J. (1991). Creating artificial neural networks that generalize. *Neural Networks*.

[16] Schwarzer G., Vach W., Schumacher M. (2000). On the misuses of artificial neural networks for prognostic and diagnostic classification in oncology. *Statistics in Medicine*.

[17] Zealand C. M., Burn D. H., Simonovic S. P. (1999). Short term streamflow forecasting using artificial neural networks. *Journal of Hydrology*.

[18] Yeh I.-C. (1998). Modeling of strength of high-performance concrete using artificial neural networks. *Cement and Concrete Research*.

[19] Coulibaly P., Anctil F., Bobée B. (2000). Daily reservoir inflow forecasting using artificial neural networks with stopped training approach. *Journal of Hydrology*.

[20] Pomerleau D. A. (1991). Efficient training of artificial neural networks for autonomous navigation. *Neural Computation*.

[21] Samanta B., Al-Balushi K. R., Al-Araimi S. A. (2004). Bearing fault detection using artificial neural networks and genetic algorithm. *Soft Computing*.

[22] Gómez H., Kavzoglu T. (2005). Assessment of shallow landslide susceptibility using artificial neural networks in Jabonosa River Basin, Venezuela. *Engineering Geology*.

[23] Conforti M., Pascale S., Robustelli G., Sdao F. (2014). Evaluation of prediction capability of the artificial neural networks for mapping landslide susceptibility in the Turbolo River catchment (northern Calabria, Italy). *Catena*.

[24] Qi-Jun Zhang Q. J., Gupta K. C., Devabhaktuni V. K. (2003). Artificial neural networks for rf and microwave design-from theory to practice. *IEEE Transactions on Microwave Theory and Techniques*.

[25] Olden J., Joy M., Death R. (2004). An accurate comparison of methods for quantifying variable importance in artificial neural networks using simulated data. *Ecological Modelling*.

